# Conformational Stability Analyses of Alpha Subunit I Domain of LFA-1 and Mac-1

**DOI:** 10.1371/journal.pone.0024188

**Published:** 2011-08-31

**Authors:** Debin Mao, Shouqin Lü, Ning Li, Yan Zhang, Mian Long

**Affiliations:** 1 Key Laboratory of Microgravity, Institute of Mechanics, Chinese Academy of Sciences, Beijing, P. R. China; 2 National Microgravity Laboratory, Institute of Mechanics, Chinese Academy of Sciences, Beijing, P. R. China; 3 Center of Biomechanics and Bioengineering, Institute of Mechanics, Chinese Academy of Sciences, Beijing, P. R. China; University of South Florida College of Medicine, United States of America

## Abstract

β_2_ integrin of lymphocyte function-associated antigen-1 (LFA-1) or macrophage-1 antigen (Mac-1) binds to their common ligand of intercellular adhesion molecule-1 (ICAM-1) and mediates leukocyte-endothelial cell (EC) adhesions in inflammation cascade. Although the two integrins are known to have distinct functions, the corresponding micro-structural bases remain unclear. Here (steered-)molecular dynamics simulations were employed to elucidate the conformational stability of α subunit I domains of LFA-1 and Mac-1 in different affinity states and relevant I domain-ICAM-1 interaction features. Compared with low affinity (*LA*) Mac-1, the *LA* LFA-1 I domain was unstable in the presence or absence of ICAM-1 ligand, stemming from diverse orientations of its α_7_-helix with different motifs of zipper-like hydrophobic junction between α_1_- and α_7_-helices. Meanwhile, spontaneous transition of LFA-1 I domain from *LA* state to intermediate affinity (*IA*) state was first visualized. All the *LA*, *IA*, and high affinity (*HA*) states of LFA-1 I domain and *HA* Mac-1 I domain were able to bind to ICAM-1 ligand effectively, while *LA* Mac-1 I domain was unfavorable for binding ligand presumably due to the specific orientation of S144 side-chain that capped the MIDAS ion. These results furthered our understanding in correlating the structural bases with their functions of LFA-1 and Mac-1 integrins from the viewpoint of I domain conformational stability and of the characteristics of I domain-ICAM-1 interactions.

## Introduction

β_2_ integrins, as important cellular adhesion molecules, play critical roles in a variety of pathophysiological processes, such as inflammation and tumor metastasis [1], [2]. Binding of the two members of β_2_ integrin, lymphocyte function-associated antigen-1 (LFA-1 or α_L_β_2_) and macrophage-1 antigen (Mac-1 or α_M_β_2_), to their common ligand intercellular adhesion molecule-1 (ICAM-1) mediates leukocyte-endothelial cell (EC) adhesion in inflammation cascade [3], [4], [5]. How do the two β_2_ integrins work together during this process? While a prevailing view had emphasized for years that LFA-1 and Mac-1 cooperatively promoted leukocyte firm adhesion, their distinct roles recently attracted much attention especially when an indispensable step so-called intraluminal crawling was added to the paradigm [6]. For neutrophils, LFA-1 mainly mediates the slow rolling and firm adhesion and Mac-1 governs the following intraluminal crawling [6], [7], [8]. For monocytes, LFA-1 supports the firm adhesion and crawling in un-stimulated condition while Mac-1 is responsible for TNF-α-stimulated crawling [4], [9]. For lymphocytes, multiple functional states of LFA-1 with different affinities are required to facilitate the cell rolling, adhesion, and crawling since no Mac-1 molecules are presented onto the cell surface [10], [11], [12]. It is also noticed that LFA-1 dominates the long-distance, meandering crawling for resident monocytes patrolling but Mac-1 mediates the short-distance, straight crawling for activated monocytes and neutrophils in the effective (trans-)migration [4], [6], [9]. Moreover, LFA-1 is able to interact with ICAM-1 in three known affinity states [13] but Mac-1 does not support neutrophil attachment to ECs without fMLP activation [14]. Distinct dynamics of β_2_ integrin activation and functioning are observed, e.g., LFA-1 responses within first 300 s after IL-8 stimulation whereas Mac-1 begins to be engaged in from 350 s after activation [15]. In short, LFA-1 is versatile in inflammation cascade whereas Mac-1 is a specific crawling mediator, and the two molecules differ in their activation dependence and dynamics.

Even with the distinct functionality of the two β_2_ integrins, they possess the high similarity in structure [2], [16], [17]. On one hand, the two molecules have the identical β_2_ subunit that is critical in transmitting the allostery bi-directionally [2]. This subunit involves I-like domain pocket capturing the bottom conservative glutamate of α_7_-helix of α subunit I domain [18], [19], [20], downward displacement of α_7_-helix of I-like domain, swinging out of hybrid domain [21], [22], extending on the knee to translocate laterally β subunit lower leg, and exerting forces on cytoskeleton by β subunit cytoplasmic domain binding to talin or kindlins [23], [24]. On the other hand, the two molecules have highly similar α_L_ and α_M_ subunits sharing 34% identical amino acids (calculated using Basic Local Alignment Search Tool (BLAST)). An I (inserted) domain of about 180–190 residues is located on the top of each α subunit. Both the α subunit I domains contain a metal ion-dependent adhesion site (MIDAS) for ligand binding, adopt Rossmann fold with 7 α-helices surrounding 6 central β-strands [16], [25], and share similar allosteric pathways by relating MIDAS re-orientation to α_7_-helix downward movement, which facilitates the capture by I-like domain pocket and transfers the conformational change to β subunit [25], [26]. Currently it is hard to correlate their distinct functionality to their highly-similar structure and allosteric patterns. One possible way to bridge the gap is to elucidate the structural bases at microscopic scale. Since the allosteric pathway of the two molecules starts from or ends at their α subunit I domain or the ligand binding domain, it is speculated that the conformational differences between their I domains, especially movement of the α_7_-helix should be, at least partially, responsible for the differences in their biological functions.

In the current work, we performed molecular dynamic (MD) simulations to test the structural evolution of LFA-1 and Mac-1 I domains upon existing crystal structures. Conformational stability was compared between two molecules as well as among three affinity states. Impact of ICAM-1 ligation on conformational stability and I domain-ICAM-1 interactions were also analyzed. Mechanical features of the complexes were tested using steered MD (SMD) simulations. Our results provided a new insight in the structure-function relationship for both LFA-1 and Mac-1.

## Methods

Two sets of molecular systems were employed in the MD simulations. The first set consists five I domain alone systems including high affinity (*HA*)/low affinity (*LA*) Mac-1 I domain (PDB codes of 1IDO [16] and 1JLM [26]) and *HA*/intermediate affinity (*IA*)/*LA* LFA-1 I domain (PDB codes of 1T0P [27], 1MJN/1MQ8 [25], and 1LFA [17]), which is used to evaluate the conformational stability. The second set is composed of five I domains from the first set ligated respectively with ICAM-1, which is used for elucidating the impact of ligand presence on I domain conformational stability as well as the binding strength of integrin-ligand complex. Since Mn^2+^ ion does not exist in physiological condition and is always referred as an activator [13], [28], [29], we replaced it by Ca^2+^ ion in the MIDAS of *LA* Mac-1/LFA-1 to avoid ionic activation possibility. *IA* LFA-1-ICAM-1 complex was adopted from the crystal structure with D1 domain of residue ID 1 to 85, and *HA*/*LA* LFA-1-D1 complexes were obtained by aligning *HA*/*LA* LFA-1 I domain to *IA* LFA-1 I domain. Noting the sequence and structure similarity between LFA-1 and Mac-1 I domains and between ICAM-1 D1 and D3 (residue ID 186 to 284) domains [30], *HA*/*LA* Mac-1-D3 complexes were constructed by aligning *HA*/*LA* Mac-1 I domain and D3 domain (PDB code of 2OZ4 [31]) onto *IA* LFA-1 I domain and D1 domain, respectively, using VMD [32] Plugin MultiSeq [33] software.

Each simulation system was built up by solvating the target molecule(s) into a rectangular water box and neutralized with ∼100 mM Na^+^ and Cl^−^ ions. NAMD program [34] with CHARMM22 all-atom force field [35] was used for the simulations. An integration time step of 1 femtosecond (*fs*) and the periodic boundary conditions were applied in the simulations. A smooth (10–12 Å) cutoff and the Particle Mesh Ewald (PME) method were employed to calculate van der Waals forces and full electrostatics, respectively. 300 *K* heat bath was manipulated under Langevin thermostat, and 1 *atm* pressure was controlled by Nosé-Hoover Langevin piston method. Prior to the equilibration process, energy minimization with 10000 steps of fixed backbone atoms followed by additional 10000 steps with all atoms free, and system heating from 0 to 300 *K* at 30 *K* increment every 5 picosecond (*ps*) were performed. 10 nanosecond (*ns*) or longer equilibration was performed for each system.

SMD simulations were also conducted to unbind the LFA-1/Mac-1-ICAM-1 complex [36]. Complex conformations resulted from the above equilibrations served as the initial conformation for SMD simulations. Here N-, C-, or N-/C-terminal C_α_ atoms of I domain were fixed independently and constant force of 200, 800, 1200 or 1600 piconewton (pN) was applied on C-terminal C_α_ atom of ICAM-1 D1 or D3 along the vector from the fixed atom or geometry center to the pulled end. All the simulation set-up was summarized in Table 1.

**Table 1:** Summary of simulation set-up.

				Free equilibration (duration (*ns*)×runs)		SMD simulations (force (*pN*)×runs)		
System	PDB code	WT/Mutation	Ligand	Alone	Ligated	N-	C-	N-&C-
*HA* Mac-1	1IDO	WT	ICAM-1 D3	10×3	10×1; 20×1	200×1; 800×4; 1200×1; 1600×1	800×1	800×1
*LA* Mac-1	1JLM	WT	ICAM-1 D3	10×6	10×4; 20×1; 30×2	200×1; 800×4	800×1	800×1
*HA* LFA-1	1T0P	K287C/K294C	ICAM-1 D1	10×3	10×2	200×1; 800×4; 1200×1; 1600×1	800×1	800×1
*IA* LFA-1	1MJN/1MQ8	L161C/F299C	ICAM-1 D1	10×3	10×2	800×2	-	-
*LA* LFA-1	1LFA	WT	ICAM-1 D1	10×11; 30×2	10×10; 20×1; 30×2	200×1; 800×14; 1200×2; 1600×2	800×1	800×4

*HA*: high affinity; *IA*: intermediate affinity; *LA*: low affinity; WT: wild type.

Geometry parameters were defined to represent the characteristics of conformational change. Root mean squared deviation of entire I domain (global RMSD) was used to quantify I domain stability, and average RMSD of each residue over time was employed to identify the unstable amino acids within I domain. Aligning the stable core residues of trajectory conformations to that of crystal structure of *HA*, *LA* (for both LFA-1 and Mac-1), or *IA* (only for LFA-1) I domain respectively, the RMSD of α_7_-helix was used to probe if the trajectory tends to switch among different affinity states. MIDAS residues [2] were regarded within the primary coordination sphere if the side-chain oxygen atom was close to the metal ion by 3.5 Å and within secondary sphere if not. Complex lifetime was defined from SMD simulations as the time interval required to separate the MIDAS ion away from side-chain oxygen of D229 of D3 or E34 of D1 around 10 Å. VMD program was used for data analysis and conformation presentation [32].

Simulations were conducted on the DeepComp 7000 supercomputer at the Computer Network Information Center, Chinese Academy of Science. Altogether, 690-*ns* MD and 783-*ns* SMD simulations were performed in this work, and spent 5,795 h on 64 compute nodes with two 3.0 GHz quad-core Intel Xeon processors.

## Results

### Conformational stability of LFA-1/Mac-1 I domains

**Figure 1:**
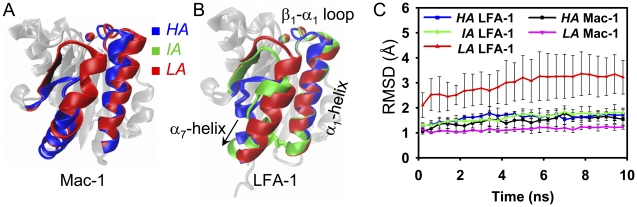
Conformational difference of LFA-1/Mac-1 I domains in different affinity states. Crystal structures of Mac-1 (*A*) and LFA-1 (*B*) were superposed, respectively, in three affinity states of *HA* (*blue*), *IA* (*green*), and *LA* (*red*). Metal ion in MIDAS (*vdW presentation*), β_1_-α_1_ loop, and α_1_- and α_7_-helices (*newcartoon presentation*) are highlighted, and other parts are presented in *transparent silver newcartoon* for clarity. The arrow directs the movement of α_7_-helix in activated allostery. Disulfide bonds introduced into the β_6_-α_7_ loop of *HA* LFA-1 and between α_1_ and α_7_-helix C-terminals of *IA* LFA-1 I domains are shown in *licorice*. All backbone atoms RMSD (global RMSD in brief) evolutions of five equilibrated I domains relative to their respective crystallized reference conformation are presented in (*C*), and *error bars* are the standard deviation (SD) of ten runs of *LA* LFA-1 and three runs of other four systems.

The crystal structures of *HA*/*LA* Mac-1 I domains and *HA*/*IA*/*LA* LFA-1 I domains were superposed in Figs. 1*A* and 1*B*, respectively. Two states of *HA* and *LA* Mac-1 and one state of *LA* LFA-1 structures are defined for wild-type molecules while two states of *HA* and *IA* LFA-1 structures stem from the mutated molecules which are stabilized by a disulfide in β_6_-α_7_ loop and a disulfide locking C-terminals of α_1_ and α_7_-helix, respectively. It is noted that both the disulfide bonds distort the α_7_-helix conformation. Two common features were observed for the structure of *IA* and *HA* states by comparing with *LA* state: MIDAS re-orientation with α_1_-helix inward movement and the distinct α_7_-helix axially downward shift [2], [26] (Figs. 1*A*–*B*). Thus, we first conducted the equilibration simulations to test their conformational stabilities. Stable conformations were visualized for the four structures except of *LA* LFA-1 I domain. To confirm this interesting observation, the simulation was repeated ten times for *LA* LFA-1 and three times for each of the other four. Global RMSDs relative to their respective crystal structures were calculated and averaged for each system. RMSD of *LA* Mac-1 I domain yielded as small value as ∼1 Å (*purple*) and that of *HA* Mac-1 and *HA*/*IA* LFA-1 I domains retained <2 Å (*black*, *blue* and *green*), while it fluctuated from 2 to 4 Å for *LA* LFA-1 (*red*) (Fig. 1*C*).

**Figure 2:**
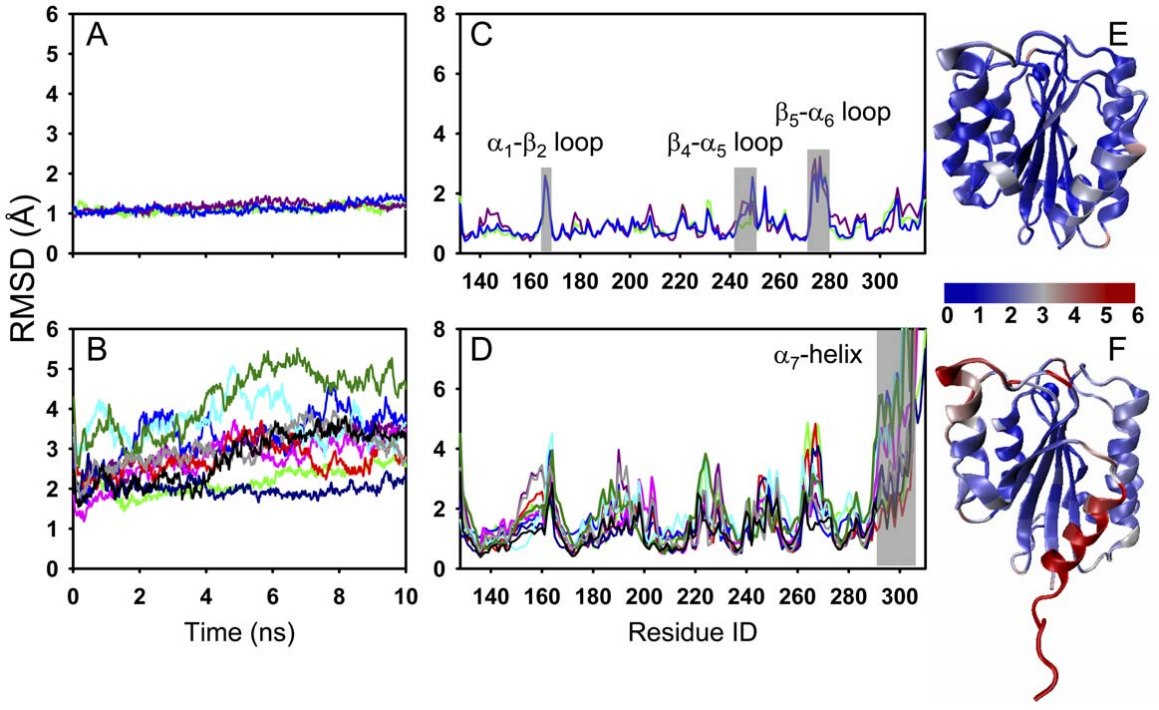
Conformational stability comparison between *LA* LFA-1 and *LA* Mac-1 I domains. Global RMSDs (*A*, *B*) and average RMSDs of each residue over time (*C*, *D*) are presented for three equilibration runs of *LA* Mac-1 I domain (*A*, *C*) and ten runs of *LA* LFA-1 I domain (*B*, *D*), respectively. Slightly flexible regions of α_1_-β_2_, β_4_-α_5_ and β_5_-α_6_ loops for *LA* Mac-1 I domain are labeled in *C*, and the most flexible region corresponding to α_7_-helix of E293 to K305 for *LA* LFA-1 I domain are labeled in *D*. *Colored lines* are denoted as the different trajectories and *grey stripe(s)* indicates those residues with remarkable or moderate conformational flexibility. Stability difference of each residue is illustrated for *LA* Mac-1 (*E*) and *LA* LFA-1 (*F*) I domains, using *changed color* from *blue* to *red* with corresponding RMSD value from 0 to 6 Å in their respective structures.

To further understand the intramolecular bases of structural instability of *LA* LFA-1 I domain, global RMSD of each trajectory and average RMSD of each residue were compared between *LA* LFA-1 and *LA* Mac-1. It was found that *LA* Mac-1 state remained stable with a small RMSD of 0.9–1.5 Å in three repeats (Fig. 2*A*) while RMSD for *LA* LFA-1 state was much higher (1.2–5.5 Å) and dramatically diverse from one to another simulation (Fig. 2*B*). Average RMSD of each residue exhibited similar trends as global one did. In fact, all the residues for *LA* Mac-1 state yielded low RMSD values (Figs. 2*C* and 2*E*) except of those moderate fluctuations around α_1_-β_2_ loop (L164-S167), β_4_-α_5_ loop (D242–G251), and β_5_-α_6_ loop (V271–S277) (*grey stripes* in Fig. 2*C*). By contrast, all the residues of *LA* LFA-1, except of those around central β-strands and α_4_-helix, seemed to be unstable (Figs. 2*D* and 2*F*), especially for MIDAS residues and α_7_-helix (E293–K305; *grey stripe* in Fig. 2*D*). RMSDs of α_7_-helix residues were significantly high (1.3–14.1 Å) and remarkably fluctuating in repeated simulations, implying that there exists the diversified conformations. Together, the conformational stability is distinct between two β_2_ integrin I domains and from one state to another, of which *LA* LFA-1 state is most likely unstable.

### Conformational diversity of LA LFA-1 I domain

**Figure 3:**
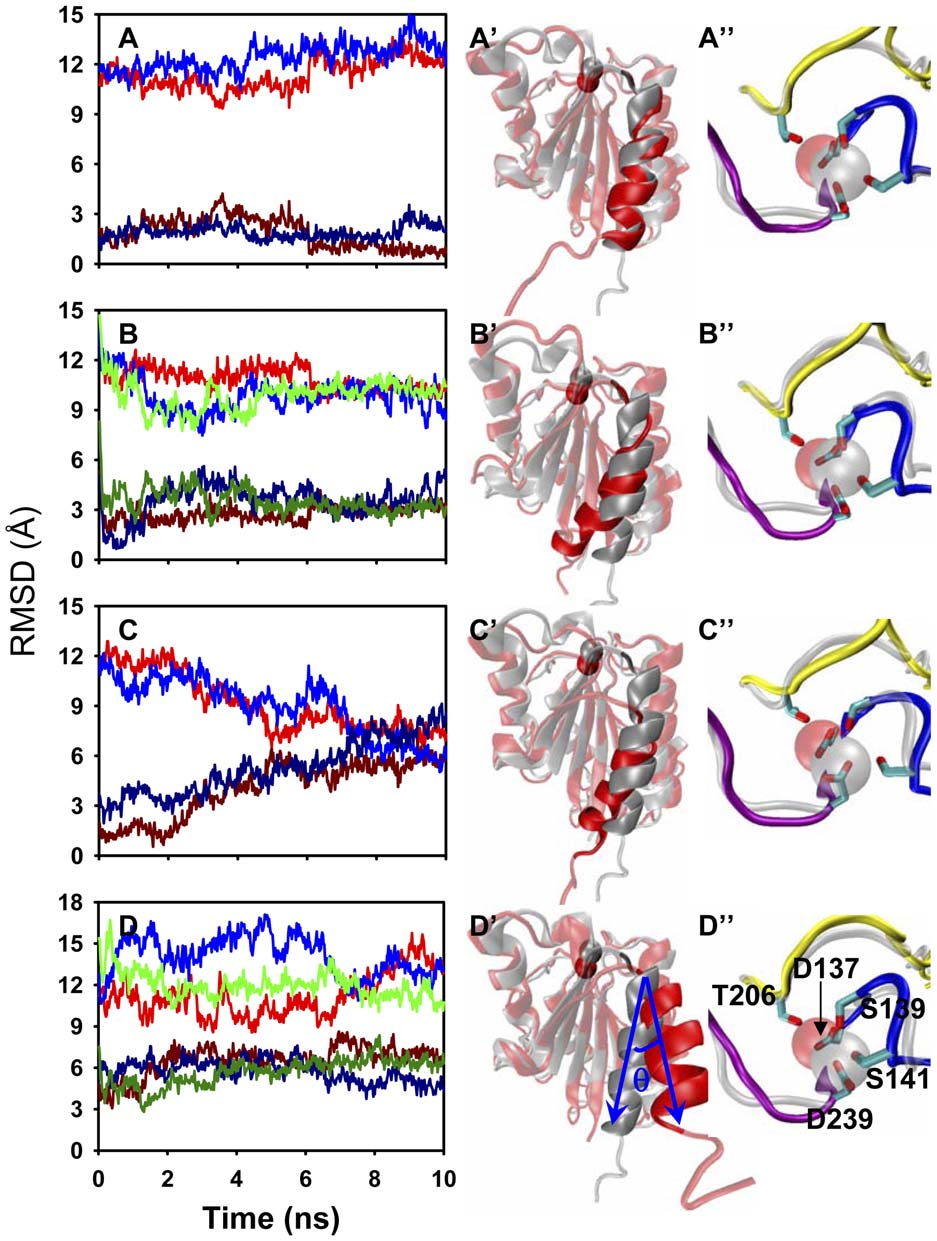
Multiple patterns of *LA* LFA-1 I domain conformation from ten equilibrations. *Left column* (*A*–*D*) denotes the time course of α_7_-helix RMSD evolution for each trajectory relative to crystal structures of *HA* (*red*, *blue* and *green lines*) or *LA* (*dark red*, *dark blue* and *dark green lines*) I domain with the stable core regions aligned. One *colored line* and the corresponding *dark-colored line* are paired for one simulation run. *Middle column* (*A*′–*D*′) exhibits the typical conformational change of MIDAS ion (*vdW* in *opaque*) and α_7_-helix (*newcartoon* in *opaque*) by superposing the end-point snapshot (*red*) with *LA* crystal structure (*silver*). The swing-out angle is demonstrated in (*D*′). *Right column* (*A*″–*D*″) illustrates the corresponding equilibrated MIDAS conformation of β_1_-α_1_ loop (*blue*), α_3_-α_4_ loop (*yellow*), and β_4_-α_5_ loop (*purple*) and the crystal structure (*silver*), which are zoomed out from *middle column*. Key residues presented with *licorice* are labeled in (*D*″) with side-chain oxygen atom in *red*. (See also Figure S1.)

Next we analyzed those diversified conformations observed in *LA* LFA-1 I domain equilibrations. Here the core residues of equilibrated snapshots were aligned to that of crystal structure of *HA*/*IA*/*LA* LFA-1 I domain, respectively, and RMSD of α_7_-helix was calculated with time which is used to define the movement of α_7_-helix. Ten repeated simulations were classified into four categories: 1) α_7_-helix stabilized in *LA* state (as observed from two of ten trajectories). Here RMSD was low when aligning equilibrated snapshot to *LA* LFA-1 crystal structure (*dark red* and *dark blue lines*), high when aligning to *HA* LFA-1 structure (*red* and *blue lines*) (Fig. 3*A*), and intermediate when aligning to *IA* LFA-1 structure (4–6 Å below that of *HA* LFA-1; *lines not shown for clarity*), which implied that the equilibrated α_7_-helix (*red newcartoon in opaque*) retained similar conformation as that of *LA* crystal structure (*silver newcartoon in opaque*) (Fig. 3*A′*). 2) α_7_-helix swung in and moved downward slightly (Fig. 3*B′*) (from three of ten trajectories). RMSDs relative to *LA* (*dark-colored lines*) or to *HA* (*colored lines*) reference exhibited a transition phase with time to approach each other followed by a stable phase at >6 *ns* (Fig. 3*B*). These conformations were different from the established *HA*/*IA*/*LA* crystal structures, and more and longer simulations are needed to confirm whether they are stable ones. 3) α_7_-helix moved downward significantly (from two of ten trajectories). This movement resembles much to the state transition allostery of I domain with a descending phase of RMSDs to *HA* reference (*colored lines*) and an ascending phase to *LA* reference (*dark-colored lines*) (Fig. 3*C*). One simulation run was extended to 30 *ns*, and the resulted steady-going phase in the last 20 *ns* indicated that the allosteric conformation was stable (Fig. *S*1). Unlike the forced or ligand-induced α_7_-helix downward-movement previously described [20], [22], the equilibrations reported here first visualized the spontaneous conformational change of α_7_-helix with a remarkable displacement of 8–11 Å for each residue (Fig. 3*C′*). 4) α_7_-helix swung outwards (from three of ten trajectories). Here RMSDs were significantly high and fluctuated from one to another run of simulation (Fig. 3*D*). Defining the angle of α_7_-helix swing-out by variation of the vector along C_α_ atom of K305 to C_α_ atom of E293, 30° to 45° rotations were observed for the equilibrated α_7_-helix (Fig. 3*D′*), which are much smaller than that found in a crystal structure (106°) [37].

While α_7_-helix presented multiple patterns of conformational change, corresponding MIDAS conformation re-orientated similarly. It was found from the ten simulations that the ion in MIDAS (*vdW*, *silver* and *red* for crystal and equilibrated structures, respectively) moved inwards about 2.1 to 2.8 Å (Figs. 3*A′*–*D′*). The coordination between the ion and MIDAS residues altered in the same way (Figs. 3*A″*–*D″*). In crystal structures, S139, S141, and D239 coordinate the ion in the primary coordination sphere and D137 and T206 in the secondary sphere. During the equilibrations, S141 moved backward to secondary sphere, and D137 and T206 moved forward to coordinate the ion tightly (residues shown in *licorice*).

### Allostery from LA to IA LFA-1 I domain

**Figure 4:**
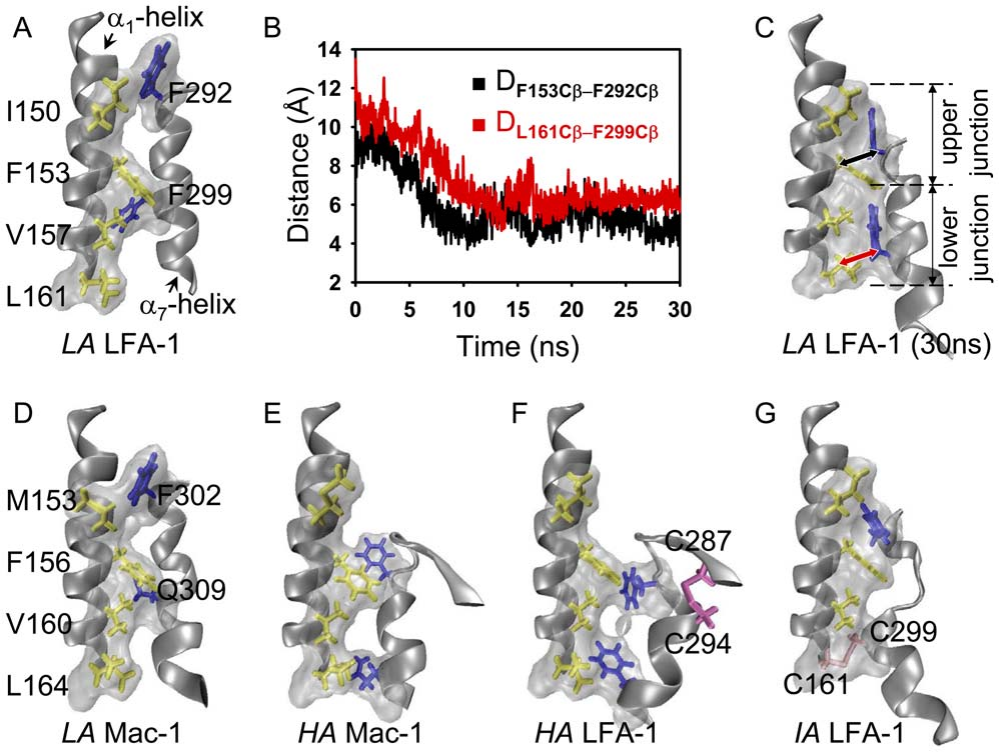
Zipper-like hydrophobic junction between α_1_ and α_7_-helices. Key residues involved in the junction formed in LFA-1 I domain are labeled as I150, F153, V157 and L161 (*yellow licorice*) locating at α_1_-helix and F292 and F299 (*blue licorice*) at α_7_-helix (*A*). Typical distance change of D_F153Cβ-F292Cβ_ and D_L161Cβ-F299Cβ_ were calculated from an extended 30-*ns* equilibration for *LA* LFA-1 I domain, as seen in Fig. *S*1 (*B*). The formed upper and lower junctions are illustrated at the end-point of 30-*ns* equilibration, resulted from the movements of F292 and F299 to the directed positions (*arrows*) (*C*). Hydrophobic junction conformation for five crystal structures of *LA*/*HA* Mac-1 and *HA*/*IA*/*LA* LFA-1 I domains are presented in (*D*), (*E*), (*F*), (*G*), and (*A*), respectively. Corresponding key residues involved in Mac-1 I domain hydrophobic junction are labeled in (*D*) and mutated disulfide bonds in *HA*/IA LFA-1 I domains are marked in (*F*) and (*G*), respectively. (See also Figure *S*2, *S*3.)

It was noticed that four categories of conformational states derived from *LA* LFA-1 I domain. The first reserved its original *LA* state (Fig. 3*A*), the forth might be artificial due to the spatial repulsion of neighboring domains (Fig. 3*D*), and the second was not proved by any crystal structures (Fig. 3*B*). The most striking is the third one that underwent a large downward movement of α_7_-helix (Fig. 3*C*). The resulted low α_7_-helix RMSD value to *IA* or *HA* LFA-1 crystal structure implied the transition to *IA* or *HA* state (Fig. *S*1). To further understand the structural features of the third category of equilibrated *LA* LFA-1 I domain conformations, the conjunction between α_1_- and α_7_-helices was tested and a novel zipper-like hydrophobic junction was identified. Here, the side-chains of F292 and F299 (*blue* in *licorice*) in α_7_-helix orientated downwards and inserted between I150 and F153 and between V157 and L161 (*yellow* in *licorice*) of α_1_-helix, respectively (Figs. 4*A* and 4*C*). Fig. 4*A* shows the conformation in crystal structure of *LA* LFA-1 and Fig. 4*C* shows what it looks like after 30-*ns* equilibration. C_β_-atom distances from F153 to F292, D_F153Cβ-F292Cβ_, and from L161 to F299, D_L161Cβ-F299Cβ_, defined to quantify the extent of the junction formation, decreased within first 10-*ns* duration followed by a steady phase in another 20-*ns* equilibration (Fig. 4*B*), which is consistent with the evolution of the α_7_-helix RMSDs (Fig. *S*1).

Conformations of those hydrophobic residues were compared among different Mac-1 or LFA-1 I domain states. Three types of zipper-like hydrophobic junction were found in the five crystal structures: the first was defined as an inadequately-zippered one where the α_7_-helix F292/F302 and F299/Q309 residues located apart in which F292/F302 wandered over the zipper head I150/M153 residues and F299/Q309 over the zipper stalk V157/V160 residues respectively in both *LA* LFA-1 and Mac-1 (Figs. 4*A* and 4*D*); the second was denoted as a over-zippered one where α_7_-helix underwent a dramatic downward movement and translocated F292/F302 residues around respective F153/F156, and F299/Q309 residues to the zipper tail L161/L164 residues in both *HA* LFA-1 and Mac-1 (Figs. 4*F* and 4*E*); the third was illustrated as a properly-zippered one where the large downward movement of α_7_-helix enforced F292 and C299 (mutated from F299) interacting strongly with their counterpart residues of α_1_-helix and closed the hydrophobic core perfectly (Fig. 4*G*). It was evident that same hydrophobic upper junction formed in equilibrated *LA* LFA-1 I domain as mutated *IA* LFA-1 I domain crystal structure while the difference was found in the lower junctions which were locked by hydrophobic interaction for the former and by a disulfide bond for the latter (Figs. 4*C* and 4*G*). In fact, the introduction of a disulfide bond resulted in a distinct upper junction in *HA* LFA-1 as compared with that in *HA* Mac-1 (Figs. 4*E* and 4*F*). A supplemental simulation by breaking up the disulfide bond in *IA* LFA-1 *via* resetting the mutated residues back to the original ones, using SWISS-MODEL workspace [38], also indicated that the large-sized side-chain of F299 pushed the lower half α_7_-helix away to rejoin with the upper half and form the properly-zippered junction (Fig. *S*2), as the one shown in Fig. 4*C*.

Time courses of the hydrophobic junction were quantitatively presented by the distance evolving between typical residues of *HA*/*LA* Mac-1 and *HA*/*IA*/*LA* LFA-1 I domains during at least 10-*ns* simulations (Fig. *S*3). The results indicated that three states have distinct hydrophobic junctions with different typical residue distances: the *HA* (*A*, *C*) and *LA* (*B*, *E*) states yielded large distances for both Mac-1 (*A*, *B*) and LFA-1 (*C*, *E*) with respective over-zippered and inadequately-zippered hydrophobic junctions, while the *IA* LFA-1 (*D*) state had small distances with properly-zippered hydrophobic junction. Four categories of *LA* LFA-1 yielded different hydrophobic junctions with different evolving of key residue distance (*E*–*H*) (Fig. *S*3). Together, these analyses support that two distinct features of hydrophobic residues exhibited: different states in same β_2_ integrin member share different hydrophobic junction conformations and the same state yields the similar hydrophobic junction in different members. And these results also indicate that the third category of equilibrated conformations of *LA* LFA-1 I domain acts as *IA* LFA-1 I domain and the allostery is able to take place spontaneously.

### Impact of ICAM-1 ligation on conformational stability

**Figure 5:**
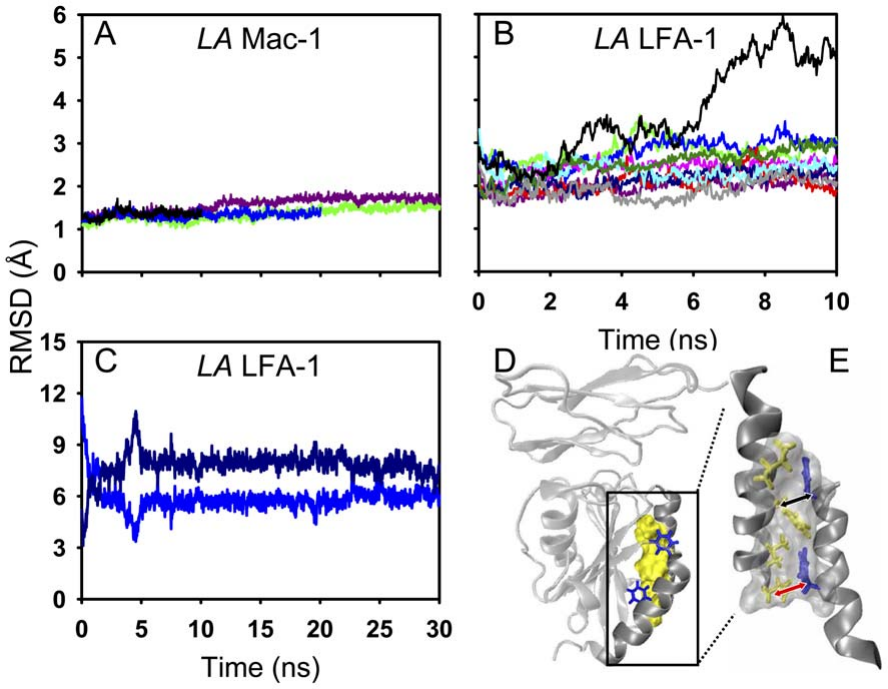
Impact of ICAM-1 ligation on conformational stability of *LA* Mac-1/LFA-1 I domain. Global RMSD evolution of I domain of *LA* Mac-1-ICAM-1 D3 (*A*) or *LA* LFA-1-ICAM-1 D1 complex is presented from four or ten repeated runs (*colored lines*), respectively. α_7_-helix RMSDs relative to *LA* (*dark blue*) or *HA* (*blue*) LFA-1 I domain reference are illustrated for a typical simulation run of *LA* LFA-1-ICAM-1 D1 complex (*C*). Corresponding hydrophobic junction at the end-point of 30-ns snapshot is displayed in front view (D) with yellow surf for α1-helix key residues and blue licorice for α7-helix key residues and in side view (E) with same presentation as shown in Fig. 4. (See also Figure S4.)

The above simulations of LFA-1/Mac-1 I domain alone indicated that *LA* LFA-1 presented the conformational instability and the spontaneous allostery of state transition but *LA* Mac-1 retained its original state stable. Meanwhile it has also been reported that the ligand binding could induce the conformational change of α_v_β_3_ headpiece [22], [39]. To test the impact of ICAM-1 ligation on conformational stability of LFA-1 and Mac-1 I domain, we further conducted the equilibration simulations for five I domain-ICAM-1 complexes and, specifically, the repeated runs for *LA* LFA-1-ICAM-1 D1 and *LA* Mac-1-ICAM-1 D3 complex for comparison. Again, *LA* Mac-1 I domain remained low global RMSDs to its crystal reference in four simulations whereas ten runs of *LA* LFA-1 exhibited the unstable features with diverse RMSDs (Figs. 5*A*–*B*), confirming that both isolated and ligated *LA* LFA-1 states were unstable. Similar four patterns of I domain conformation, as those found for I domain alone (*cf.* Fig. 3), were also observed with different proportions in ten simulations of *LA* LFA-1-D1 complex, of which four simulations denoted to *LA* state, three to slight swing-in and downward movement of α_7_-helix, two to large α_7_-helix downward movement, and one to 60° swing-out movement of α_7_-helix (Fig. *S*4). Moreover, the resulted conformation with large α_7_-helix movement (*cf*. Figs. 3*C* and *S*2*C*) was stable with steady α_7_-helix RMSDs relative to *HA*/*LA* LFA-1 references found in an extended 30-*ns* equilibration (Fig. 5*C*). Calculated C_β_-atom distance of D_F153Cβ-F292Cβ_, (*black arrow*) and D_L161Cβ-F299Cβ_ (*red arrow*) also supported the same hydrophobic junction as that of *IA* LFA-1 (Figs. 5*D–E*). Thus, these results indicated that ICAM-1 ligation played a limited role in conformational stability and allosteric features of LFA-1 and Mac-1.

### The difference between LFA-1 and Mac-1 I domain-ICAM-1 interactions

**Figure 6:**
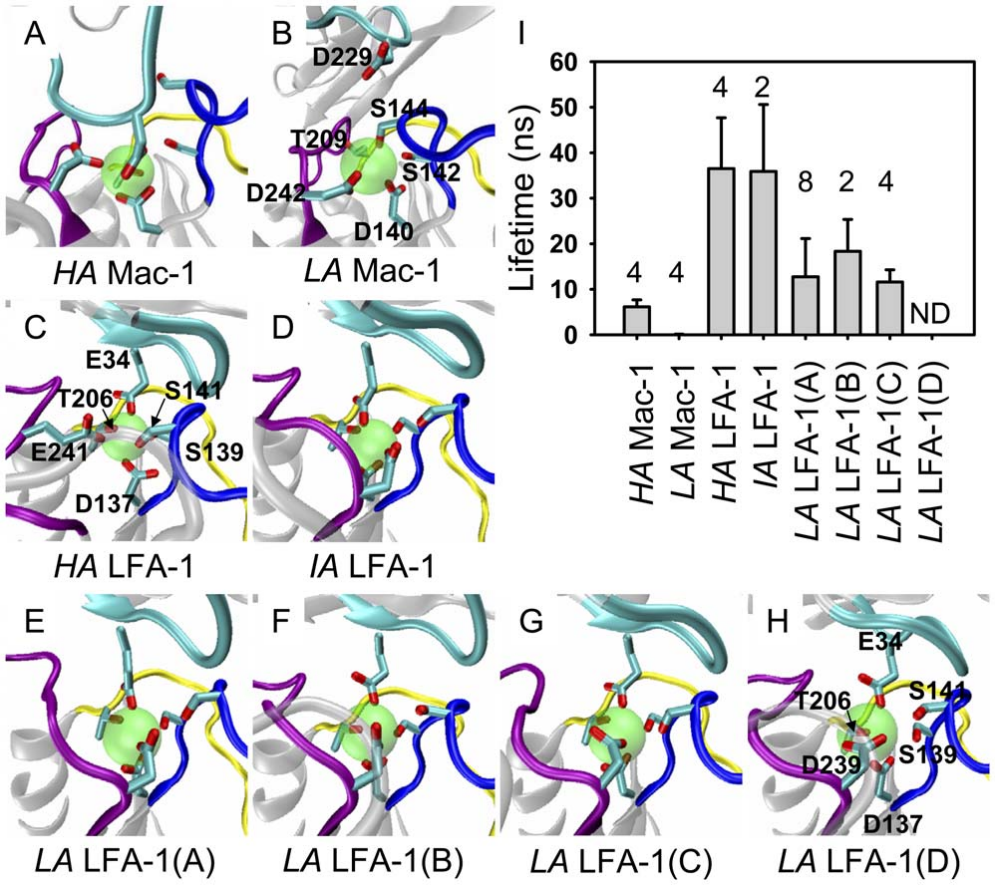
LFA-1 and Mac-1 I domain-ICAM-1 interactions. (*A*–*H*) Key interactions between I domain MIDAS ion (*vdW* in *transparent*) with the relevant residues and D1 domain E34 residue or D3 domain D229 residue. Three loops of MIDAS (denoted in *newcartoon*) are respectively colored in *blue* (β_1_-α_1_ loop), *yellow* (α_3_-α_4_ loop) and *purple* (β_4_-α_5_ loop) and the CD loop located with E34 of D1 and D229 of D3 is colored in *cyan*. Related residues are denoted in *licorice* and labeled in (*B*) for Mac-1, in (*C*) for *HA* LFA-1, and in (*H*) for *IA* LFA-1 as well as all four *LA* LFA-1 cases. (*I*) Complex lifetime is estimated, using a SMD simulation of 800-pN constant force, by pulling C-terminal C_α_ atom of ICAM-1 D1 or D3 domain along the vector from fixed atom of N-terminal C_α_ atom of I domain to the pulled end. Here four cases of *LA* LFA-1 (*A*–*D*) are denoted as those equilibrated structures of *LA* LFA-1 I domain-ICAM-1 D1 complex originated from Fig. *S*4. *Numbers* are the repeated runs and *error bars* are the SD of mean lifetime. (See also Figure S5.)

Finally we compared the difference between LFA-1 and Mac-1 I domain-ICAM-1 interactions. Detailed conformational comparison illustrated that the main difference between *HA* and *LA* Mac-1 I domain-ICAM-1 D3 domain interactions attributed to the orientation of I domain S144 side-chain. While there exists free space enabling D229 residue of D3 domain to interact readily with MIDAS ion in *HA* Mac-1 state (Fig. 6*A*), the tight interaction of S144 side-chain with MIDAS ion most likely reduced the accessibility of D229 residue (Fig. 6*B*), even though the ion located ∼2 Å more closer to D229 residue for *LA* Mac-1 than that for *HA* Mac-1 for the systems built. The ligated *HA* LFA-1 I domain yielded a distinct MIDAS conformation where β_1_-α_1_ loop (*blue in newcartoon*) pointed inwards to enable S141 residue coordinating the ion in the primary coordination sphere and the re-orientation of β_4_-α_5_ loop (*purple in newcartoon*) induced E241 substitution for D239 residue in the primary sphere (Fig. 6*C*). Interestingly, MIDAS conformations were similar for ligated *IA* (Fig. 6*D*) and *LA* LFA-1 (four cases in Figs. 6*E*–*H*) where D137 and T206 directed forwards to the primary coordination sphere and S141 pointed backwards to the secondary sphere (except for the ligated swung-out one, of which both S139 and S141 pointed backwards). The repeated simulations presented the similar MIDAS conformations and ion-D229/E34 interactions, especially for the wide type systems (Fig. *S*5), which confirmed, to some extent, the strategy of complex construction in the current work. Remarkably, the simulation was repeated for *LA* Mac-1-ICAM-1 four times from 10 to 30-*ns*, in which not even once D229 was able to break in and catch the ion, most likely due to the shielding of S144 (Fig. *S*5*B*).

We further tested the mechanical strength of the complex to illustrate if and how the conformational change of I domain would affect the binding function of β_2_ integrin to ICAM-1 ligand. Upon the end-point equilibrated structures of those five complexes, SMD simulations of 800 pN constant force were performed by pulling C-terminal C_α_ atom of ICAM-1 D1 or D3 domain along the vector from fixed atom of N-terminal C_α_ atom of LFA-1 or Mac-1 I domain to the pulled end. Since the interaction of MIDAS ion and D229 or E34 residue of D3 or D1 domain provides key resistance to external force, the complex lifetime, defined as the duration to separate MIDAS ion beyond 10 Å from D229 or E34 side-chain oxygen atom, was used to quantify the binding strength. It was found that the lifetime significantly reduced from *HA* (6.15±1.53 *ns*) to *LA* Mac-1 I domain (0.11±0.04 *ns*) (*1^st^* and *2^nd^ bars*), which is consistent to that for the shielding of S144 to abrogate ion-D229 binding (Fig. 6*I*). And comparable lifetime was found for *HA* (36.53±11.16 *ns*) or *IA* (35.89±14.70 *ns*) LFA-1 I domain to D1 domain, which was dramatically higher than those for Mac-1 I domain (*3^rd^* and *4^th^ bars*), presumably due to the introduced disulfide bonds. Three cases of *LA* LFA-1 I domain, corresponding to the first three categories shown in Fig. *S*4, yielded the intermediate lifetime values of 12.74±8.40 (*A*), 18.33±7.01 (*B*), and 11.59±2.69 *ns* (*C*), respectively (*5^th^*–7*^th^ bars*) (Fig. 6*I*). The lifetime for another case of *LA* LFA-1 I domain (*D*), the fourth category presented in Fig. *S*4, was not determined, mainly due to that the swing-out conformation of α_7_-helix may not be practical. These results indicated that all states of LFA-1 I domain were able to bind to D1 domain effectively but *LA* Mac-1 I domain was unfavorable in binding to D3 domain with poor mechanical resistance. Such the nature was further confirmed by varying the fixed end from N-terminal to C-terminal and to jointed N- and C-terminals and altering the external forces from 200 to 1600 pN. In fact, even though the favorable unfolding of α_7_-helix was visualized when only C-terminal C_α_ atoms was fixed, both the fixation of C-terminal and of jointed N- and C-terminals yielded similar feature with that from N-terminal fixation (*data not shown*), implying the fixation strategy used here is appropriate. Since applying the force of 200 pN is unable to dissociate *HA*/*LA* LFA-1-ICAM-1 and *HA* Mac-1-ICAM-1 complexes in 36-*ns* simulation but applying a force of 1200 or 1600 pN dissociates the complex too fast, considering the balance between simulation time and conformational details, most of SMD simulations employed a typical force of 800 pN. Total fifty-one SMD tests were done in different cases, as summarized in Table 1. All the tests under varied forces yield similar features of bond lifetime among five complexes (data not shown on 200, 1200, or 1600 pN). Combined with the complex lifetimes and the MIDAS conformations, the results demonstrated that MIDAS ion and related residues played the key roles in determining the characteristics of Mac-1/LFA-1 and ICAM-1 interactions.

## Discussion

This work aimed at elucidating the structural bases for distinct functionality of LFA-1 and Mac-1 by analyzing the conformational stability of their α subunit I domains of different affinity states in the absence or presence of ICAM-1 ligand. The novelty of our work lies in the following aspects: First, *LA* LFA-1 I domain is found to be more flexible than that of *LA* Mac-1 (Figs. 1*C* and 2, 3, 5) and thus, it readily possesses conformational change to implement the versatile function of LFA-1 in inflammation cascade. Second, *LA* LFA-1 I domain is able to interact stably with ICAM-1 D1 domain and resist applied force effectively (Figs. 6, *S*5). Combined with the conformational instability of I domain, one possible mechanism is proposed that *LA* LFA-1 binds ICAM-1 first and then its flexible α_7_-helix of I domain moves downward to connect with β subunit for further conformational modulation, which is crucial to cellular mechanotransduction and biological functions such as slow rolling [7], [8]. Recently published MD simulations showed that the interaction between bent α_v_β_3_ integrin and its RGD ligand could bear the pulling and then induce α_v_β_3_ to extend [40]. Third, the stubborn *LA* Mac-1 I domain is unable to open its pocket properly for ICAM-1 binding with the unfavorable orientation of S144 (Fig. 6, *S*5), supporting that Mac-1 is insufficiently functional without activation. This may also provide a hint to interpret why Mac-1 is unable to react as fast as LFA-1 does when the inflammation occurs. Together, these simulations offer important structural clues for understanding the functional differences between LFA-1 and Mac-1.

Spontaneous transition from *LA* state to *IA* state of wild type LFA-1 I domain is crucial to the conformational variations as well as biological functions of β_2_ integrin. On one hand, this is the first time to visualize the spontaneous allostery of LFA-1 I domain in MD simulations (Figs. 3, 4, 5), although it has been reported that applied forces drives the transition of *LA* to *IA* and then to *HA* of I domain [20] and that ligand binding induces the downward movement of α_7_-helix of β_3_ I-like domain [22], [39]. The minor restriction of α_7_-helix exhibited in newly-crystallized α_X_β_2_ structures indicates that I domain α_7_-helix has enough space for spontaneous allostery within whole molecule [18], so the allostery from *LA* LFA-1 to *IA* LFA-1 presented in this study is structurally feasible. Combined with the indication that resting β_3_ integrin could spontaneously extend when being solvated in hydrodynamic octylglucoside [41], this new finding provides a clue that *IA* LFA-1 with both extended legs and *IA* I domain may exist on resting leukocytes and cooperate with *LA* LFA-1 in cell adhesions as a result of the balance of multiple conformations. On the other hand, it is further observed from zipper-like hydrophobic junction of *IA* LFA-1 that the lockage of α_1_ and α_7_-helice together is a general mechanism to retain certain conformational state for integrin [20], [39]. Upon the similarity of hydrophobic junction conformations and related residues between *LA*/*HA* Mac-1 I domains and those of *LA*/*HA* LFA-1 (Figs. 4*A*, 4*D*, 4*E*, and 4*F*), it seems reasonable to predict the existence of stable *IA* state of Mac-1 I domain with a zipper-like junction similar to that for *IA* LFA-1 (Figs. 4*C*, 4*G*, and *S*2*C*). Thus, the finding of the novel *IA* state of LFA-1 I domain, which spontaneously evolved from *LA* state, furthers the understandings in structural variations.

It has long been recognized that the conformational change in different affinity states of integrin α subunit I domain lies in two major regions, one is for MIDAS and the other is for α_7_-helix, where the MIDAS conformation usually re-orientates distinctly corresponding to multiple conformational patterns of α_7_-helix. In contrast, our simulations illustrate no significant difference in MIDAS conformations for the residues reserved in *LA* state and those turned to *IA* or other states in the absence (Fig. 3) or presence (Fig. *S*4) of ICAM-1 ligation. Even though further investigations are required to elucidate the subtle difference, this speculation of conformational irrelevance between MIDAS and α_7_-helix regions seems to be reasonable, since these two regions are so flexible in *LA* LFA-1 that they may work independently in the absence of supporting domains. And the results may also attribute to the limitation of insufficiently long equilibrations and to the uncertainty of conformational transition dynamics.

In the current work, five complex systems were constructed upon high similarity in structure by aligning the target molecules to those crystallized complex available in PDB database, which worked quite well for both LFA-1 and Mac-1. All states of LFA-1 I domain binds to ICAM-1 with the vital coordination of MIDAS ion to E34 of D1 finely formed (Figs. 6*C*–*H* and *S*5*C*–*G*). Our SMD simulations of the resulted complexes indicate that the *LA* states of LFA-1 I domain with distinct conformations are able to bind to its ligand (Fig. 6*I*), which is consistent with the observation that ICAM-1 bound to LFA-1 at different conformations, including the bent one with the lowest affinity [13]. For Mac-1, *HA* state I domain is able to bind to ICAM-1 ligand effectively with the key coordination of MIDAS ion to D229 of D3 stably formed and the moderate mechanical resistance (Figs. 6*A*, 6*I*, and *S*5*A*). *LA* state I domain consistently prevents its MIDAS ion from binding to D229 of D3 domain in four 10 to 30-*ns* simulations (Figs. 6*B*, 6*I* and *S*5*B*), which resembles to the mutation of D229 that completely abrogated the binding of Mac-1 to ICAM-1 [42].

It should also be pointed out that the identity of divalent cation has insignificant impacts on I domain stability and I domain-ICAM-1 interactions for both LFA-1 and Mac-1, since the replacement of Ca^2+^ by Mg^2+^ retained the similar global RMSDs of I domain and similar I domain-ICAM-1 interaction patterns (Fig. *S*6). Such the observations are consistent with those crystal studies, in which the conformation of I domain is not altered in the presence or absence of MIDAS ion or by the replacement of MIDAS ion [43], [44], as well as with those measurements that isolated I domain is not activated by Mg^2+^ or Mn^2+^ [28], [29]. The structural bases for the effect of Mg^2+^ or Mn^2+^ activation on wild type LFA-1 but not isolated I domain require further investigations [13], [29].

In summary, the flexibility of LFA-1 I domain and the rigidity of Mac-1 I domain are basic features for the two β_2_ integrins, which are highly correlated to their allosteric pathways and biological functions. Flexible LFA-1 is usually ready for ligand binding and easy to perform state transition, while the functionality of stubborn Mac-1 requires additional cytokine signaling [14], [45]. Thus, these structural analyses suggest that LFA-1 is able to interact quickly in multiple biological functions such as cell rolling, firm adhesion, transient migration while Mac-1 seems to mediate the slow interactions mainly on crawling in inflammation cascade.

## Supporting Information

Figure S1 **α_7_-helix RMSD of a 30-** ***ns*** **equilibration of** ***LA*** **LFA-1 I domain alone when aligned the core residues to those of** ***HA*** **/** ***IA*** **/** ***LA*** **LFA-1 crystal structures.** Noting that the *green line* is 4 to 6 Å underneath the *blue line* during first 10 *ns*, as mentioned in the text, but merges together at the last 18 *ns*.
(TIF)

Figure S2 **Global RMSD of wide type** ***IA*** **LFA-1 I domain (** ***A*** **) and zipper-like hydrophobic junction between its α_1_ and α_7_-helices of the end-point snapshot (** ***B*** **and** ***C*** **).** Same presentations are shown as in Figs. 5*D* and 5*E*.
(TIF)

Figure S3 **Stability of the zipper-like hydrophobic junction.** Distance evolving between typical residues was calculated for *HA*/*LA* Mac-1 (*A*, *B*), *HA*/*IA* LFA-1 (*C*, *D*) and four cases of *LA* LFA-1 (*E*–*H*). For Mac-1, *black* and *red* lines denote D_F156Cβ-F302Cβ_ and D_L164Cβ-Q309Cβ_, respectively. For LFA-1, same presentations are shown as in Fig. 4*B*, except for the *red* line of *IA* LFA-1 denotes D_C161Cβ-C299Cβ_. Here four cases of *LA* LFA-1 (*E*–*H*) correspond to the typical equilibrations of four categories of *LA* LFA-1 I domain shown in Fig. 3.
(TIF)

Figure S4 **Multiple patterns of** ***LA*** **LFA-1 I domain conformation from ten equilibrations of** ***LA*** **LFA-1-ICAM-1 D1 complex.** Same presentations are shown as in Fig. 3.
(TIF)

Figure S5 **LFA-1/Mac-1 - ICAM-1 interactions for all repeated complex simulations.** Same presentations are shown as in Fig. 6.
(TIF)

Figure S6 **Global RMSDs of I domain equilibrated alone (** ***A*** **,** ***B*** **) or ICAM-1 ligated (** ***C*** **,** ***D*** **), and I domain-ICAM-1 interactions (** ***E*** **,** ***F*** **) for** ***LA*** **Mac-1 (** ***A*** **,** ***C*** **,** ***E*** **) and** ***LA*** **LFA-1 (** ***B*** **,** ***D*** **,** ***F*** **), for the equilibration simulations with ion substitution of Ca^2+^ by Mg^2+^ in their MIDAS site.** Same presentations are shown as in Fig. 6 for (*E*) and (*F*).
(TIF)
