# Supplementary figures and images for: Conformational Stability Analyses of Alpha Subunit I Domain of LFA-1 and Mac-1
Source: PLoS One. 2011 Aug 31;6(8):e24188. doi: 10.1371/journal.pone.0024188 (PMC3164198; doi:10.1371/journal.pone.0024188)

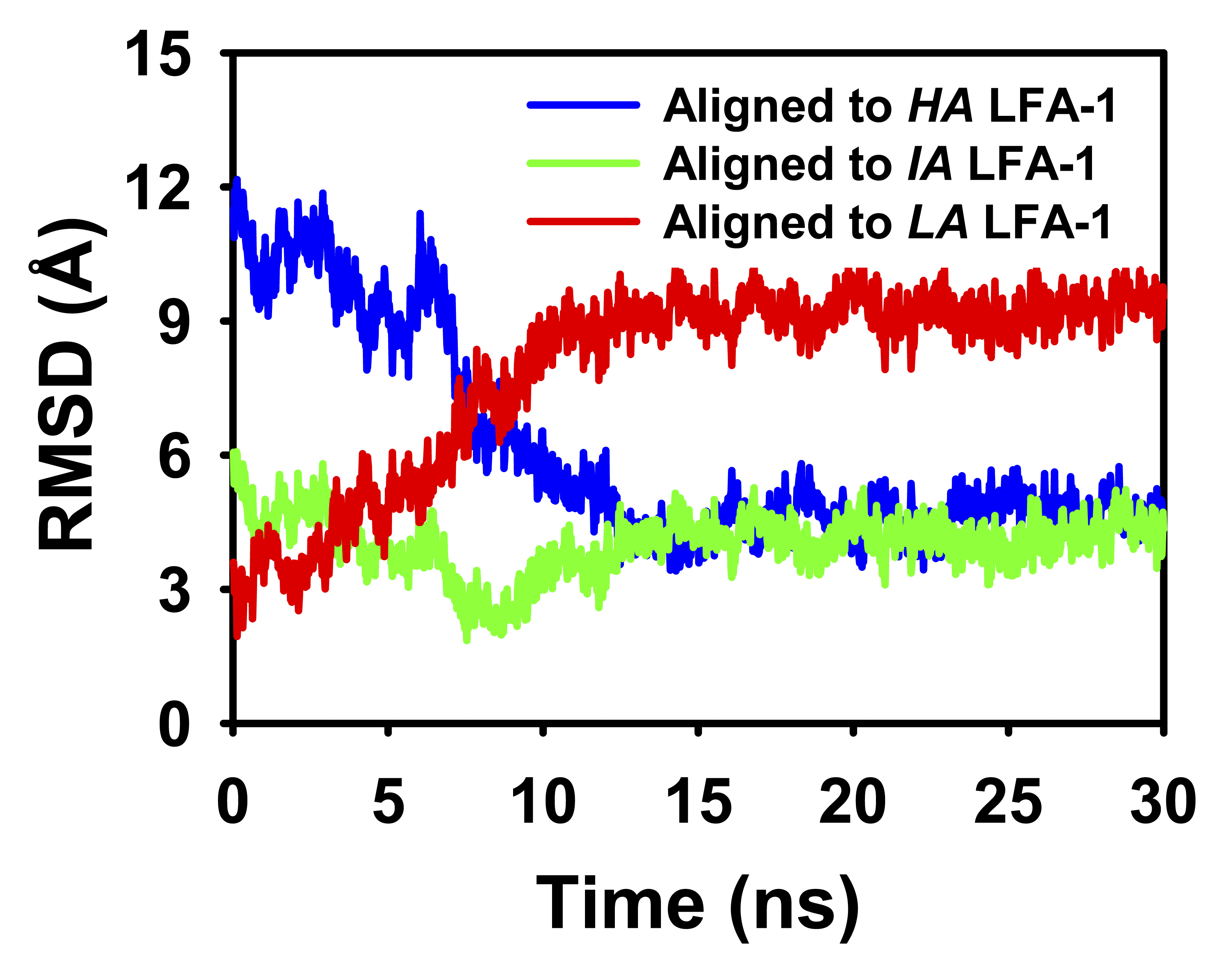

Supplement: Figure S1 — α7-helix RMSD of a 30- ns equilibration of LA LFA-1 I domain alone when aligned the core residues to those of HA / IA / LA LFA-1 crystal structures. Noting that the green line is 4 to 6 Å underneath the blue line during first 10 ns, as mentioned in the text, but merges together at the last 18 ns. (TIF) [file pone.0024188.s001.tif]

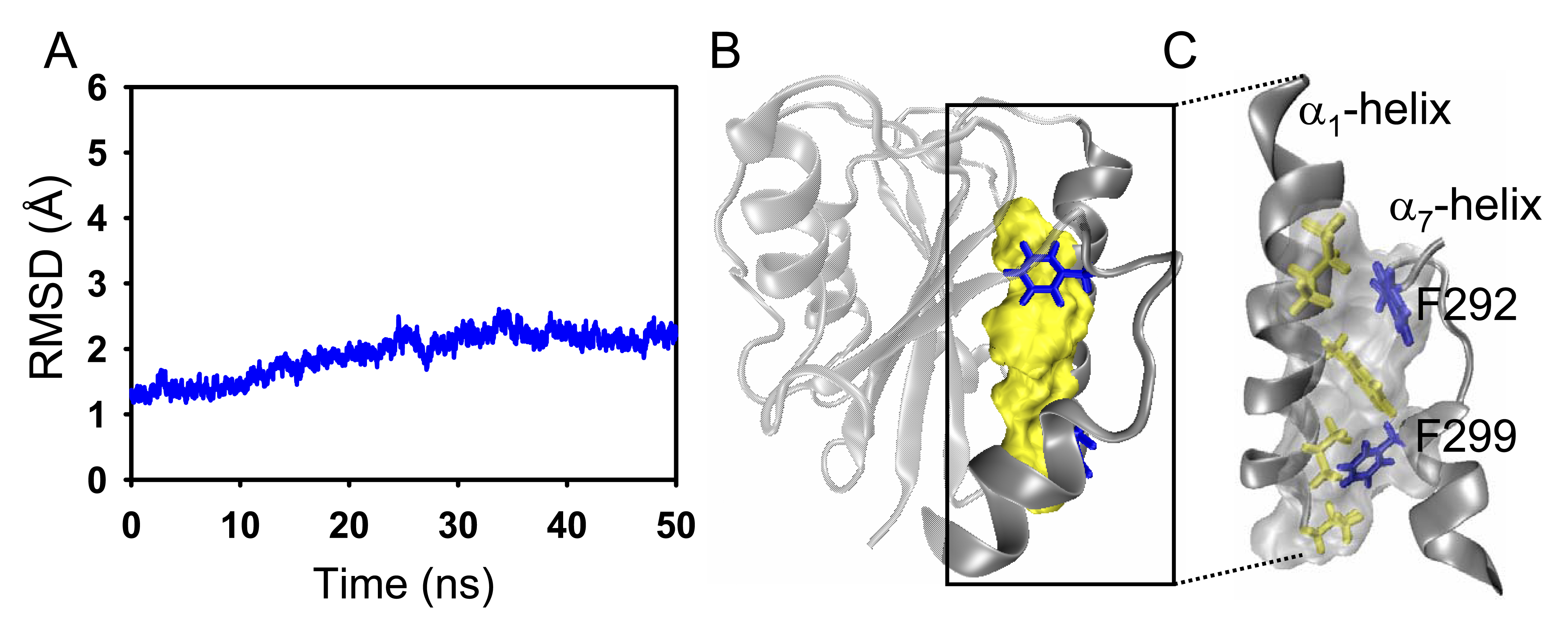

Supplement: Figure S2 — Global RMSD of wide type IA LFA-1 I domain ( A ) and zipper-like hydrophobic junction between its α1 and α7-helices of the end-point snapshot ( B and C ). Same presentations are shown as in Figs. 5D and 5E . (TIF) [file pone.0024188.s002.tif]

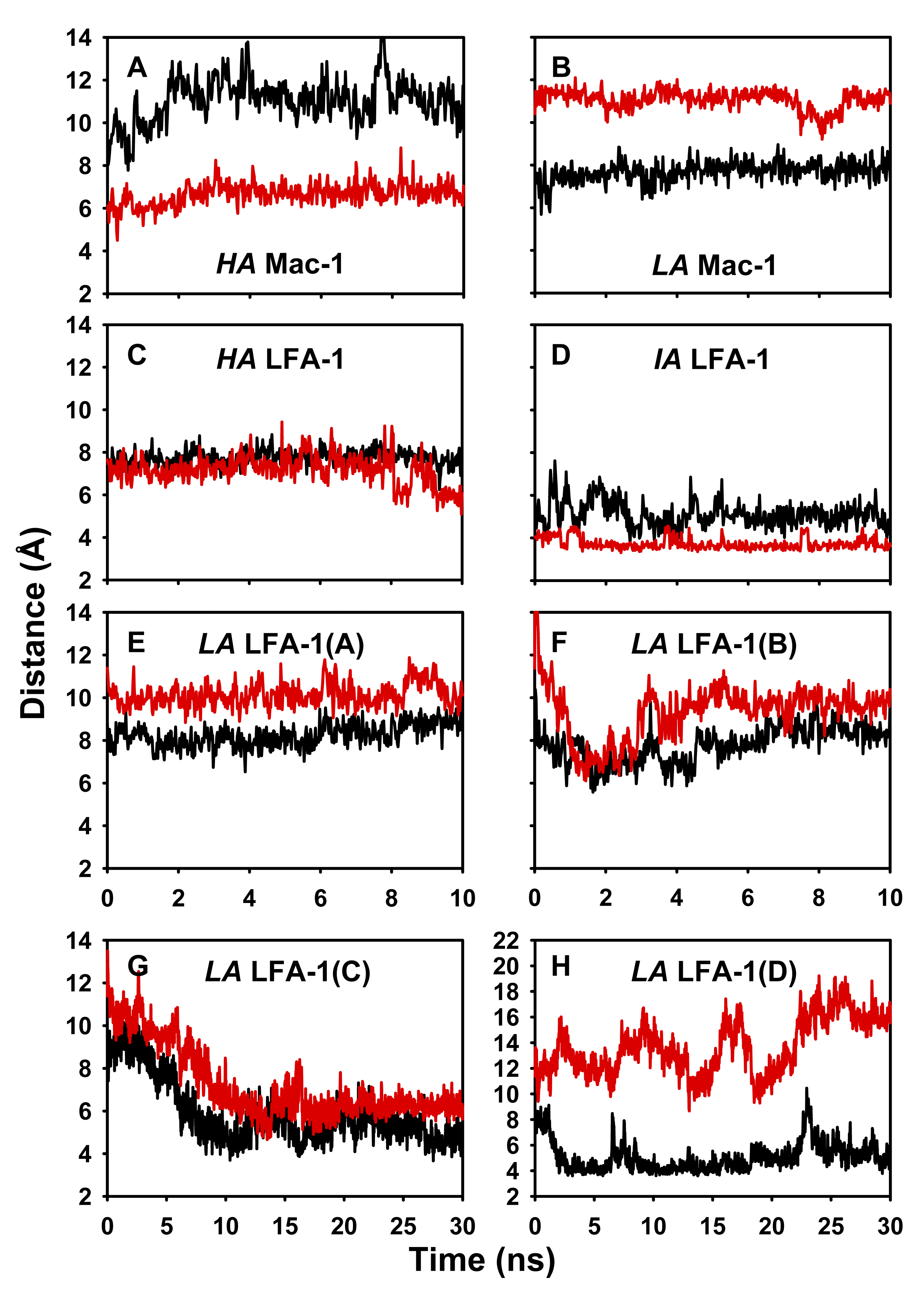

Supplement: Figure S3 — Stability of the zipper-like hydrophobic junction. Distance evolving between typical residues was calculated for HA/LA Mac-1 (A, B), HA/IA LFA-1 (C, D) and four cases of LA LFA-1 (E–H). For Mac-1, black and red lines denote DF156Cβ-F302Cβ and DL164Cβ-Q309Cβ, respectively. For LFA-1, same presentations are shown as in Fig. 4B , except for the red line of IA LFA-1 denotes DC161Cβ-C299Cβ. Here four cases of LA LFA-1 (E–H) correspond to the typical equilibrations of four categories of LA LFA-1 I domain shown in Fig. 3. (TIF) [file pone.0024188.s003.tif]

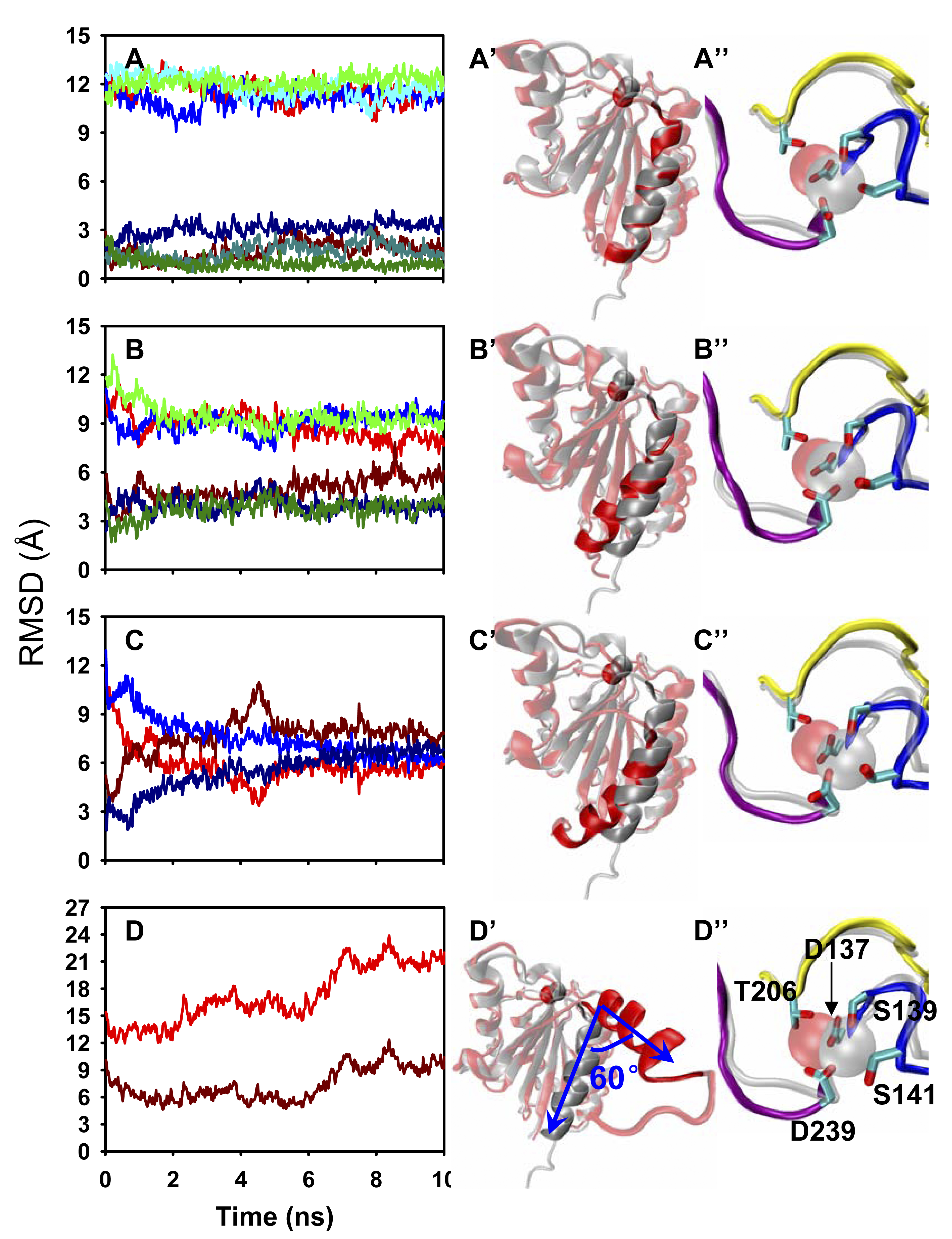

Supplement: Figure S4 — Multiple patterns of LA LFA-1 I domain conformation from ten equilibrations of LA LFA-1-ICAM-1 D1 complex. Same presentations are shown as in Fig. 3. (TIF) [file pone.0024188.s004.tif]

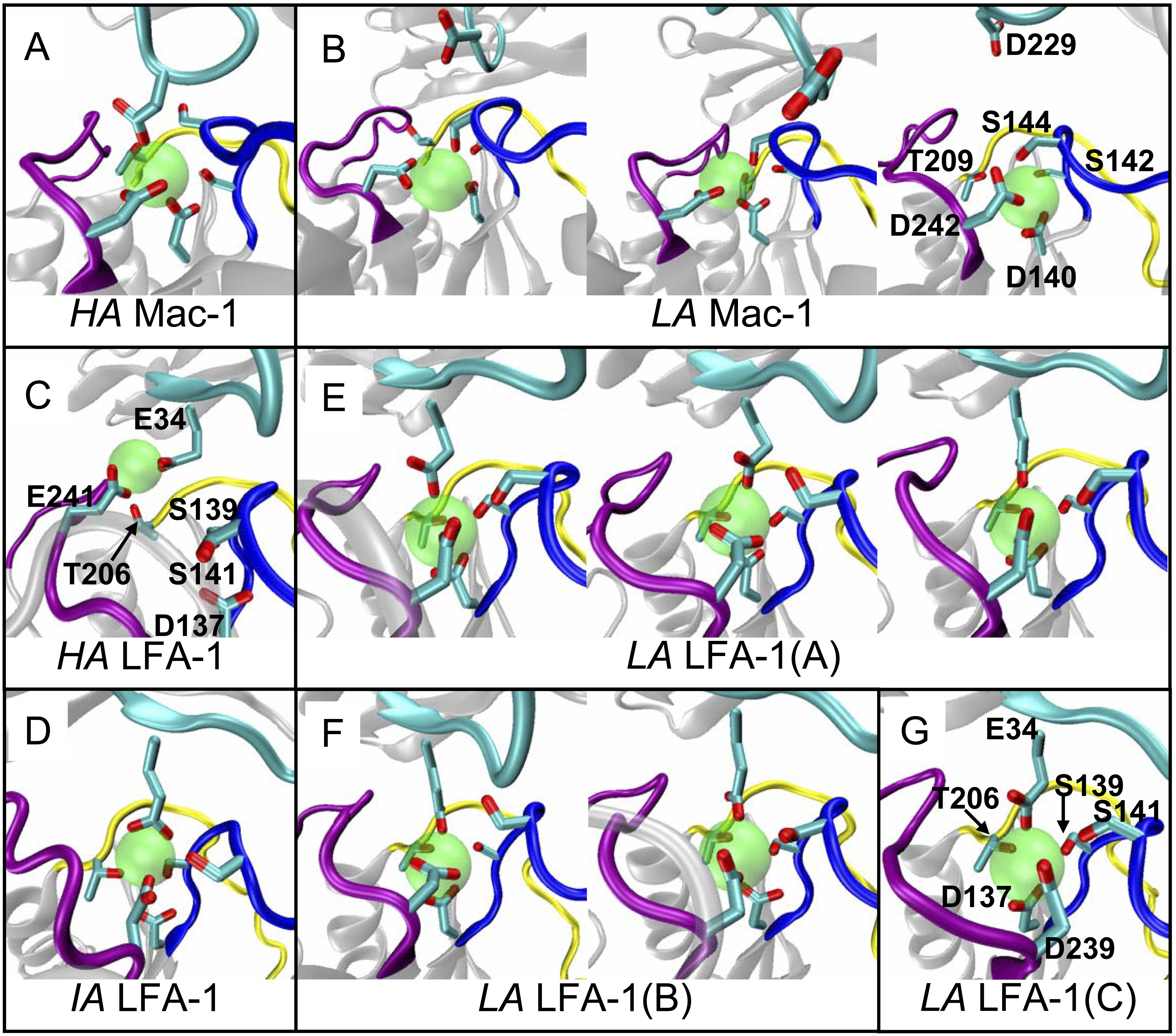

Supplement: Figure S5 — LFA-1/Mac-1 - ICAM-1 interactions for all repeated complex simulations. Same presentations are shown as in Fig. 6. (TIF) [file pone.0024188.s005.tif]

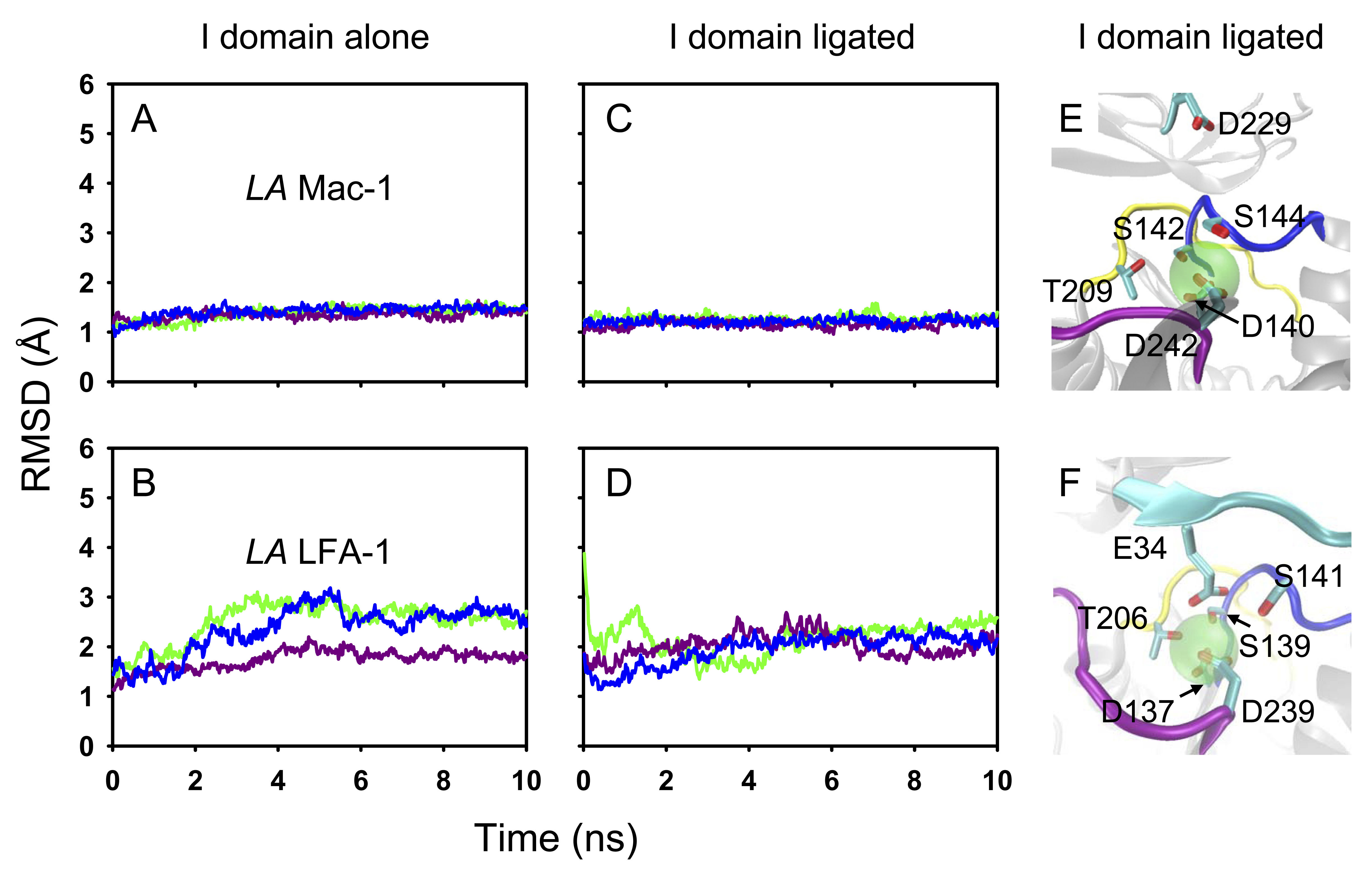

Supplement: Figure S6 — Global RMSDs of I domain equilibrated alone ( A , B ) or ICAM-1 ligated ( C , D ), and I domain-ICAM-1 interactions ( E , F ) for LA Mac-1 ( A , C , E ) and LA LFA-1 ( B , D , F ), for the equilibration simulations with ion substitution of Ca2+ by Mg2+ in their MIDAS site. Same presentations are shown as in Fig. 6 for (E) and (F). (TIF) [file pone.0024188.s006.tif]
